# Expression, purification and biological activity assessment of romiplostim biosimilar peptibody

**DOI:** 10.1186/s40199-016-0156-7

**Published:** 2016-07-11

**Authors:** Shima Fayaz, Pezhman Fard-Esfahani, Majid Golkar, Mojgan Allahyari, Sedigheh Sadeghi

**Affiliations:** Biochemistry Department, Pasteur Institute of Iran, Tehran, 1316943551 Iran; Molecular Parasitology Laboratory, Department of Parasitology, Pasteur Institute of Iran, Tehran, 1316943551 Iran; Recombinant Protein Production Department, Research and Production Complex, Pasteur Institute of Iran, Karaj, 3159915111 Iran

**Keywords:** Romiplostim, Peptibody, Immune thrombocytopenic purpura (ITP)

## Abstract

**Background:**

Romiplostim is a peptibody analogue of thrombopoietin (TPO) which regulates platelet production. This molecule consists of two main parts: Peptide sequences which like wild type TPO, mimics stimulation of TPO receptor and IgG1Fc, (Peptide + Antibody = Peptibody). This drug is used in treatment of chronic Immune Thrombocytopenic Purpura (ITP).

**Methods:**

In this project *E. coli* bacteria were transformed by a construct harboring peptibody fusion gene. This construct consisted of two repeated peptide sequences which have fused to Carboxyl group of IgG1Fc. Designed construct in *E. coli* host resulted in protein expression in cytoplasm as inclusion body. The inclusion bodies were separated, washed and after denaturation and solubilization, in the last stage the desired peptibodies were refolded and purified. The resulting peptibodies were characterized by SDS-PAGE and Western immunoblotting. The bioactivity were assessed in vivo using subcutaneous injection in mice.

**Results:**

Results showed accurate molecules were produced and purified. Also, in vivo experiment showed significant increment (more than two fold) of platelets compared to control group.

**Conclusion:**

In this study laboratory scale production of recombinant romiplostim showed proper in-vivo bioactivity. This new approach in expression and purification of this recently introduced thrombopoietin receptor agonist drug may be followed by scale up of its production to response the chronic ITP patient’s demand.

## Background

Immune thrombocytopenic purpura (ITP) is considered as an autoimmune disorder characterized by platelet deficiency due to platelet destruction and/or insufficient production. [1, 2]. Although the exact cause of ITP is not well known, mediating mechanisms in the progress of thrombocytopenia as anti-platelet antibodies and cellular (T-cell) mediated mechanisms are proven [2]. According to European studies, the incidence of ITP in adults is estimated as 1.6 to 2.25 cases per 100,000 population per year (platelet count ≤50 ×10^9^ cells/L) [3, 4]. The first step of ITP treatment includes administration of corticosteroids or intravenous immunoglobulin (IVIg). Patients who do not respond, are candidate for splenectomy. Indeed, 25 % to 30 % of patients with chronic ITP do not respond to initial treatment or splenectomy [2, 5].

First generation thrombopoietic agents were recombinant forms of human Thrombopoietin (TPO), like PEG-MGDF [6]. TPO is the endogenous ligand for the thrombopoiein receptor which is expressed on the surface of platelets and megakaryocytes and involves in stimulating thrombopoiesis [7, 8]. The expansion of these agents did not continued because of neutralizing auto-antibodies cross-reacting with endogenous TPO. Therefore, recently introduced second-generation thrombopoiesis stimulating molecules, with no sequence homology to endogenous TPO, are used [7]. Romiplostim is one of these new agents which has already completed phase III trials in primary immune thrombocytopenia and had taken FDA approved for treatment of ITP patients resistant to corticosteroid therapy and speloectomy [1]. Romiplostim is an Fc-peptide fusion protein, or peptibody. It consists of 2 identical single-chain subunits, each containing human IgG1 Fc domains linked covalently at the C-terminus to a peptide that contains 2 thrombopoietin (TPO) receptor (c-Mpl)–binding domains (4 binding sites in total) [9, 10]. It prompts the transcriptional pathways that stimulate the c-Mpl receptor, leading to improved platelet production [11, 12]. In this study we have constructed romiplostim recombinant plasmid in *E coli*, expressed and analyzed it in the laboratory scale.

## Methods

### Cloning of romiplostim gene

Nucleotide sequence of Romiplostim was retrieved from the US patent (6835809 B1) and the synthetic lyophilized construct purchased from BIOMATIK Company. Four microgram of lyophilized DNA was suspended in 40 μl nuclease-free water (final concentration 100 ng/μl) and 2 μl of suspension was transformed into *E. coli* (DH5α) according to InsTAclone PCR Cloning Kit (Thermo Scientific). The mixture was plated on LB agar (+Amp) and incubated overnight at 37 °C. Next day, some well separated single colonies were selected and inoculated in LB medium (+Amp) at 37 °C for overnight culture.

To confirm transformation, colony PCR with M13 primers were performed and the positive colonies’ plasmids were purified (GeneJET Plasmid Miniprep Kit, Thermo Scientific) and digested with *Hind* III restriction enzyme (Vivantis) overnight at 37 °C to confirm the fragment insertion.

### Subcloning of romiplostim gene in pET22b (+) expression vector

The plasmid was purified and double digested with *Bam*HI/*Nde*I restriction enzymes (Vivantis) to retrieve the subjected fragment. On the other hand, pET-22b(+) was cleaved with same restriction enzymes. The Romiplostim fragment was ligated into the pET-22b(+) using T4 DNA ligase (Fermentas), and the ligation mixture was transformed to BL21 (DE3) *E. coli*. The positive clones were selected based on the result of colony PCR with T7 universal primers.

### Expression of recombinant romiplostim

Expression of recombinant Romiplostim was done in LB broth and optimized in different times (2, 4, 6 h and overnight) with IPTG (1 mM) as inducer at 30 and 37 °C.

Five ml of media was inoculated by 500 μl of an overnight fresh culture of expression host. Induction was carried out when culture had reached to the O.D. of 0.5 at 600 nm. Zero, 2 h, 4 h, 6 h and overnight samples besides overnight sample without IPTG and overnight sample of BL21 (DE3) (without any plasmid) were centrifuged in 4°c at 13000 rpm for 5 min. Pellets were resuspended in 5× SDS-PAGE sample buffer and water boiled for 5 min. The analysis was performed by 12 % SDS-PAGE electrophoresis followed by comassie brilliant blue G-250 staining (Merck).

### Western blot analysis

The separated proteins by 12 % SDS-PAGE were transferred into nitrocellulose for 1 h at 100 V, 350 mA using wet Bio-Rad transfer system (Bio-Rad, Hercules, CA). The membrane was blocked by 2 % BSA and 0.1 % Tween 20 in 1× TBS buffer (pH 7.4) at 4 °C for overnight. Then it was incubated in 1/2000 dilution of Affinpure Goat Anti-Human IgG Fc_γ_ Fragment Specific (Jackson) for 1 h at 37 °C. Then, it was washed 4 times by 1× TBS and 1 % Tween 20 (pH 7.4) as washing buffer. It was followed by incubation in 1/1000 dillution of Rabbit Anti Sheep Ig Peroxidase conjugated (SPH 224, Avicina) for 1 h at 37 °C and washed again as mentioned. 3,3′-diaminobenzidine tetrahydrochloride (DAB) was used as substrate for visualization of the subjected protein bands in presence of hydrogen peroxide.

### Purification of romiplostim recombinant protein

#### Lysing the inclusion bodies

The cultured bacteria was centrifuged and the resulted pellet was washed in ice cold STE (0.1 M NaCl, 10 mM Tris. Cl, 1 mM EDTA) pH 8.0. The ice cold solution (50 mM Tris. Cl, 10 % Sucrose) pH 8.0, 2 ml of fresh lysozyme (10 mg/ml in 10 mM Tris. Cl [pH 8]), 0.25 M EDTA and PMSF was added to the washed pellet as lysis buffer, mixed vigorously and inverted several times on the ice surface. Cells were hold at −20 °C for 1 h. Afterward cells were lysed completely by sonication with 40 cycles: 30s on, 15 s off, at 0 °C; amplitude: 10 (MSE Soniprep 150 Plus). The lysate was centrifuged at 4 °C, 4000 rpm for 30 min. and the supernatant was discarded.

#### Washing and solubilization

The pellet was re-suspended with 6 ml wash buffer (2 M Urea, 100 mM Tris. Cl [pH 7.0], 5 mM EDTA, 2 %(w/v) Triton X100) per gram of wet weight cells and the procedure was repeated for three times. After that the pellete was re-suspended in wash buffer lacking Triton X-100 and Urea. After each washing step, the supernatant was centrifuged at 4 °C, 4000 rpm for 30 min. The pellet was re-suspended in 10 times solubilization buffer (8 M Urea, 50 mM Tris, 8 mM DTT [pH 10]) for 1 h in the shaker at room temperature [13].

#### Refolding

This stage was down according to US patent (6835809 B1. In brief. the solubilized mixture was diluted slowly 20 times into Tris 50 mM, Arginine 160 mM, Urea [1-2 M] and Cystein [3-5 mM], pH 8.5. The mixture was stirred at 4 °C, overnight. The solution was concentrated about 10 fold with Amicon Ultra-15 Centifugal Filter (cut-off: 50 kDa). Afterward it was diluted three folds in Tris 10 mM and Urea 1.5 M, pH 9. The refolding step was done.

#### Affinity chromatography

The recombinant Romiplostim mixture was passed over Protein A Sepharoose (SIGMA-ALDRICH) by Sodium Phosphate buffer 20 mM (pH 7.0) to result in Fc region binding. The Fc mediated bonded peptibody was eluted by sodium acetate buffer 20 mM (pH 4.0). The purified Romiplostim was run in to 12 % SDS-PAGE electrophoresis and immunoblotting was carried out. Bio-Rad Protein Assay was performed.

#### Biological activity

Twenty normal female BALB/c mice (7–9 weeks of age) were divided into control and case groups. Recombinant Romiplostim (50 μg/kg) and normal saline (0.2 mL/mice) were subcutaneously injected into case and control groups, respectively, on day 0. The whole blood factors were evaluated by hematology analyzer (Sysmex KX-21) on days 0, 4, 7, 17 through preorbital sinus sampling.

## Results and discussion

### Construction of expression vector

**Fig. 1:**
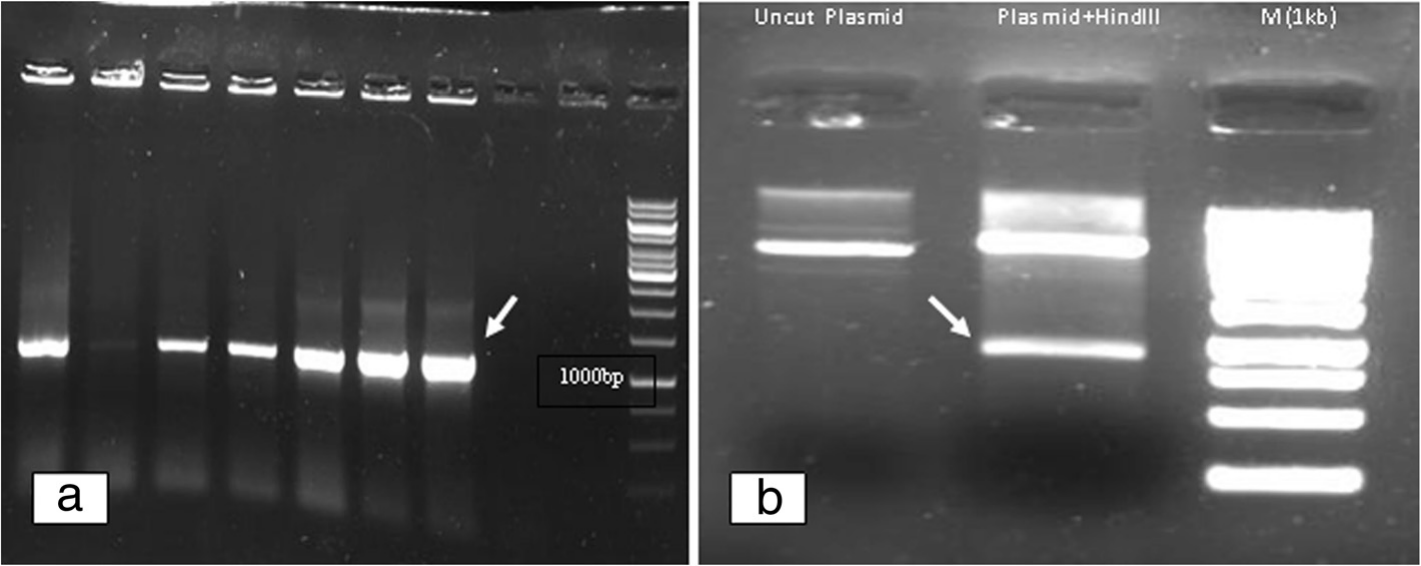
**a** Colony PCR with M13 primers, Romiplostim fragment (1000 bp) is shown; **b** The plasmid containing inserted fragment (Romiplostim) treated by *HindIII* restriction enzyme, Romiplostim fragment is indicated

**Fig. 2:**
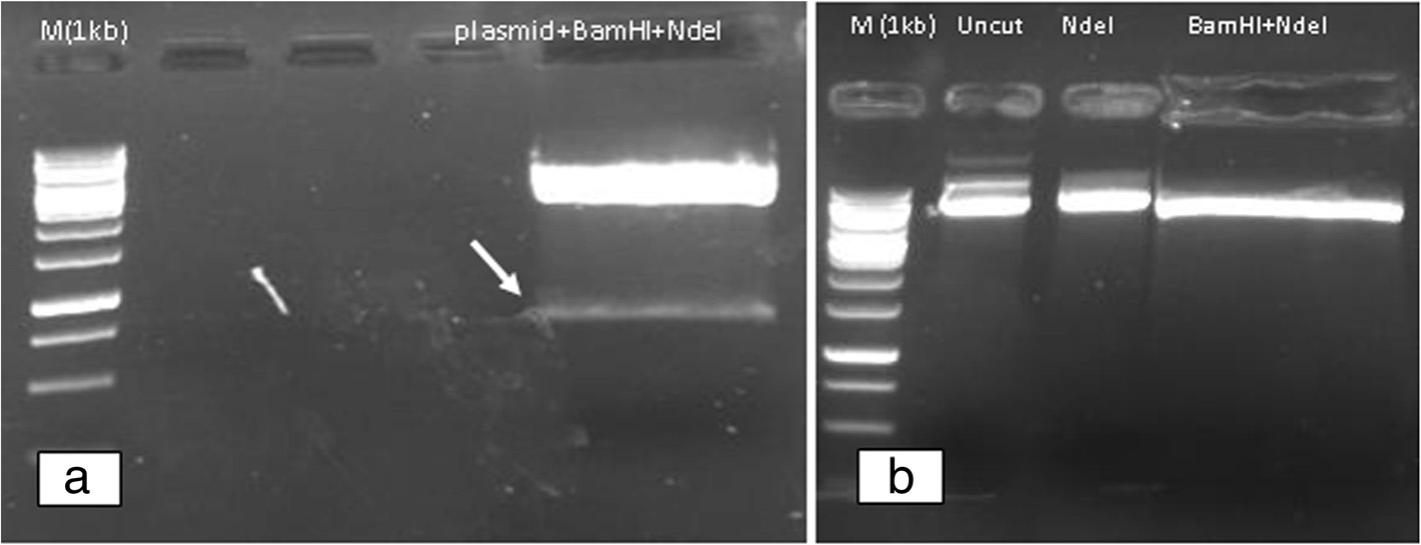
**a** Double digestion of the Romiplostim plasmid with *BamHI/NdeI*, Romiplostim fragment is identified; **b** Double digestion of pET-22b(+) with *BamHI/NdeI*

**Fig. 3:**
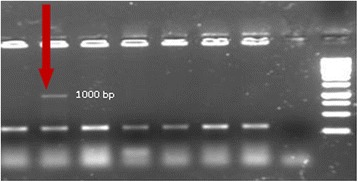
Colony PCR with T7 primers, Romiplostim fragment is indicated

There are many hosts used for the production of recombinant protein but the preferred choice is *E. coli* due to its easier culture, short life cycle, well known genetics and easy genetic manipulation. Production of recombinant protein in *E. coli* is less costly than using other hosts and the handling is also easier [14]. Therefore the synthetic fragment was successfully transformed into DH5α *E. coli* competent cells. Colony PCR with M13 primers beside *Hind*III restriction enzyme assay, showed expected bands at 1000 and 800 bp, respectively (Fig. 1a, b). In this study insoluble form of Romiplostim as inclusion body was assessed and pET22b(+) vector was used. This vector harbor secretion signal and 6xHis fusion sequences, which were omitted from the vector and 6xHis sequences has situated after the stop codon. Amplified fragment and pET-22b(+) plasmid vector were purified and digested with *Bam*HI*/Nde*I. The fragments were gel purified and subjected to ligation (Fig. 2a, b). After transformation into BL21 (DE3), colony PCR with T7 universal primers was carried out for the retrieved correct colons. The expected size was about 1000 bp (Fig. 3).

### Expression analysis

**Fig. 4:**
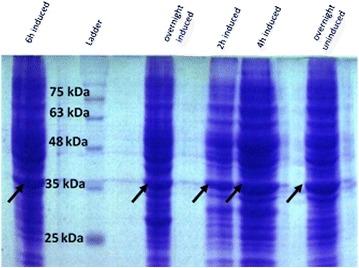
SDS-PAGE analysis of 2 h, 4 h, 6 h and overnight induced with 1 mM IPTG beside overnight uninduced of recombinant Romiplostim. High expression of Romiplostim inclusion body in uninduced recombinant Romiplostim was observed

**Fig. 5:**
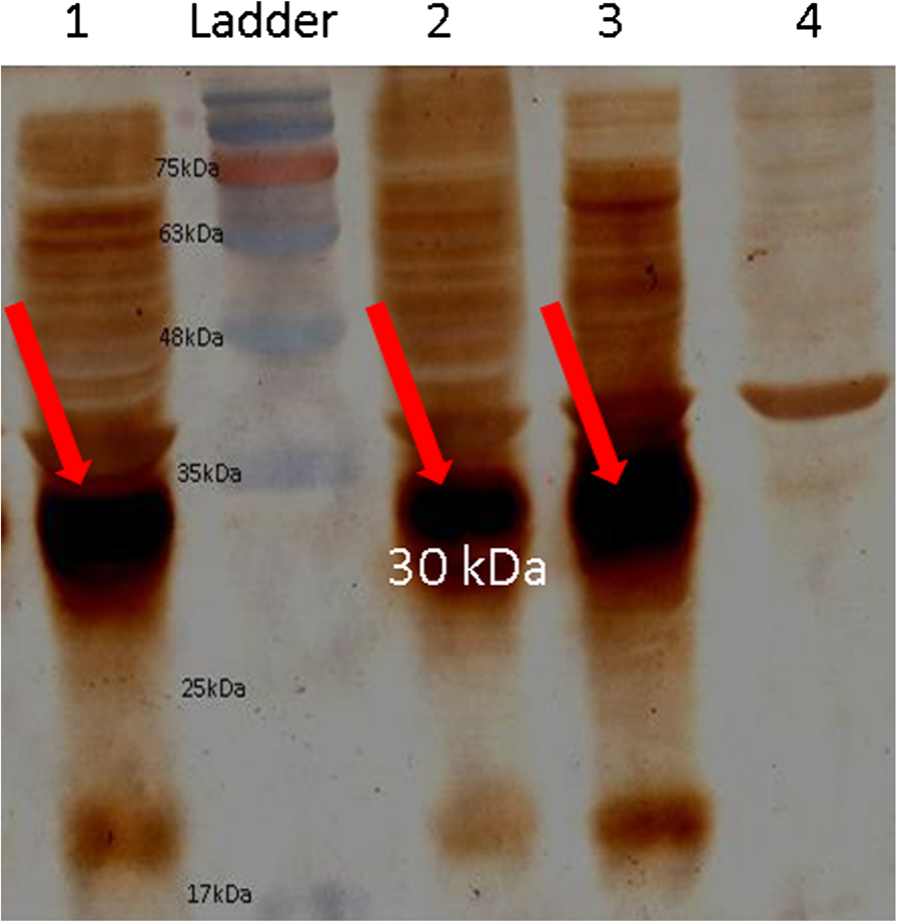
Immunoblottig pattern comparison of expressed recombinant protein in induced and uninduced cells; lane 1: 6 h induced cells by IPTG (1 mM), lane 2: Overnight induced cells by IPTG (1 mM), lane 3: Overnight uninduced cells and lane 4: Overnight BL21 (DE3) without any plasmid (nonspecific band is seen)

Expressed recombinant protein of induced and uninduced cells, was compared (Fig. 4), high expression of Romiplostim inclusion body in the absence of IPTG was a great cost effective point. In Western blot, Fc_γ_ carrier domain of the recombinant protein was reactive to goat anti-human IgG Fc_γ_ as a primary antibody, followed by peroxidase conjugated rabbit anti-sheep IgG. Sharp and distinct Romiplostim recombinant protein band in 30 kDa was in accordance with the calculated molecular weight of Romiplostim single strand (Fig. 5).

### Refolding and purification of recombinant romiplostim

**Fig. 6:**
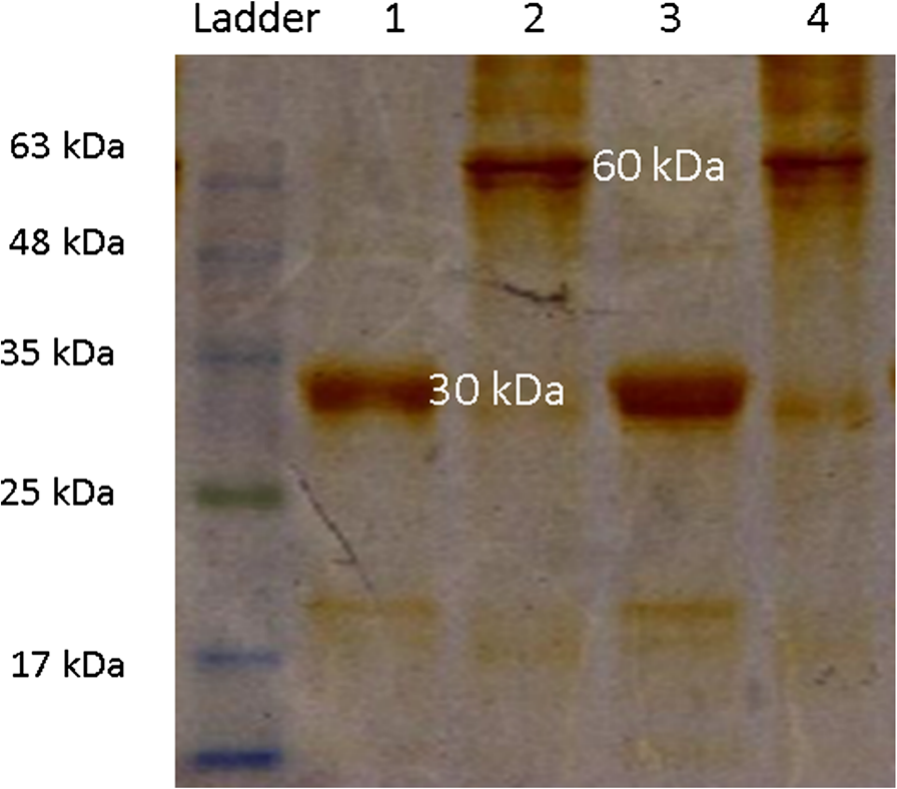
Immunoblottig pattern of the purified recombinant Romiplostim. Lane 1: Monomeric structure of the recombinant Romiplostim (30 kDa), disulfide bonds are reduced with 2ME; Lane 2: Dimeric structure of recombinant Romiplostim (60 kDa), 2ME was omitted of the loading buffer. The findings showed accurate molecules were produced and purified

In contrast to US patent (6835809 B1) which researchers recruited two step Ion Exchange chromatography, in the present study we used protein A affinity chromatography system to reduce purification stages. In order to perceive refolded and purified recombinant Romiplostim by protein A affinity chromatography, immunoblotting was performed (Fig. 6); 2ME was omitted of the loading buffer, so 60 kDa protein band was shown which was related to the quaternary structure of this protein. Besides, 30 kDa protein band in the form of denaturated protein was observed (loading buffer contained 2ME). These findings showed accurate molecules were produced and purified.

### Biological activity

**Fig. 7:**
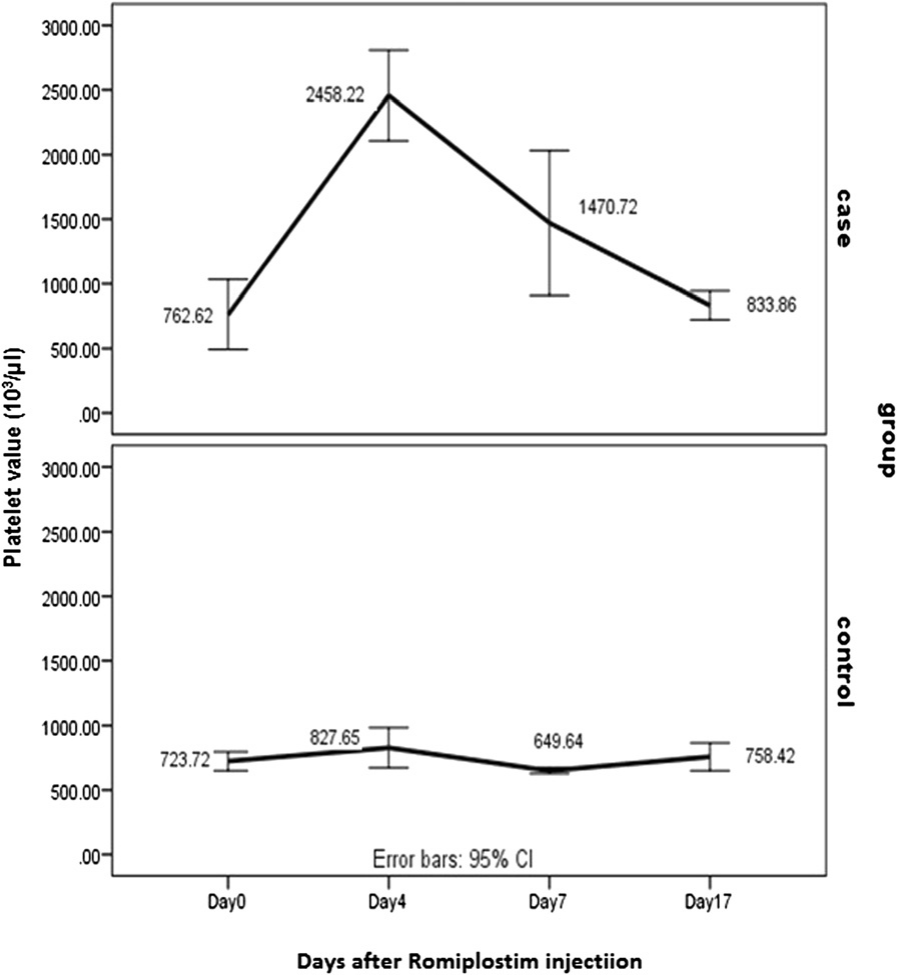
Comparison of platelet counts (10^3^/μl) between recombinant Romiplostim Injected Group and Control Group on days 0, 4, 7 and 17. On day 0 Recombinant Romiplostim (50 μg/kg) and normal saline (0.2 mL/mice) were subcutaneously injected into case and control groups, respectively. Mean platelet counts were assayed on days 0, 4, 7, 17 through preorbital sinus sampling. As it is shown in the recombinant Romiplostim treated group, platelet value has increased nearly more than 2 folds to the control group on day 4, and it returned to day 0 level on day 17. Error bar (95 % CI): Platelet counts in 10 BALB/c mice in each case and control group

Our data indicated that platelet value has increased approximately more than 2 fold in the recombinant Romiplostim treated group to the control group on day 4, and it returned to day 0 level on day 7 and 17 (Fig. 7). Other blood factors remained unchanged on days 0, 4, 7 and 17 (it is showed in attach file). As it was expected, platelet increase was transient and returned to the normal range after several days, it was proved in the previous study too [15]. So weekly injection in keeping platelet count is required. Laboratory scale production of Romiplostim may be followed by scale up of its production to response the chronic ITP patients’ demands.

## Conclusion

In this study, we evaluated a new approach for expression and purification of this recently introduced thrombopoietin receptor agonist drug. Using pET vector, we produced recombinant Romiplostim in *E. coli*, and showed proper in-vivo bioactivity. However, further characterization of Romiplostim e.g. by cicular dichroism is needed. In addition, dose dependence compression of the bioactivity of this recombinant protein with Romiplostim (N-Plate) which is in clinical use, is required.
